# Public Policy Responses to Address the Mental Health Consequences of the COVID-19 Pandemic: Evidence From Chile

**DOI:** 10.3389/fpubh.2021.590335

**Published:** 2021-09-29

**Authors:** Matias Irarrazaval, Pablo Norambuena, Cristian Montenegro, Olga Toro-Devia, Belen Vargas, Alejandra Caqueo-Urízar

**Affiliations:** ^1^Department of Mental Health, Ministry of Health, Santiago, Chile; ^2^Millenium Institute for Research in Depression and Personality, Santiago, Chile; ^3^School of Public Health, Faculty of Medicine, Universidad de Chile, Santiago, Chile; ^4^Wellcome Centre for Cultures and Environments of Health, University of Exeter, Exeter, United Kingdom; ^5^Instituto de Alta Investigación, Universidad de Tarapacá, Arica, Chile

**Keywords:** mental health, health policies, COVID-19, pandemic, Latin America

## Abstract

**Objectives:** This paper reviews the mental health policies that have been implemented in Chile in response to the COVID-19 pandemic and the international context of countries' responses. Even before the start of the pandemic, there were significant barriers to access mental health services in Chile, coupled with a scenario of nationwide social unrest and protests that questioned the legitimacy of public institutions; now the rapidly worsening outbreaks of COVID-19 are exacerbating the pre-existing mental health crisis.

**Methods:** We conducted a bibliometric and content analysis of the Chilean mental health public policies implemented during the COVID-19 pandemic and then compared these policies with international experiences and emerging scientific evidence on the mental health impact of pandemics.

**Results:** Our analysis of the policies identifies five crucial points of action developed in Chile: (i) an established framework to address mental health in emergency and disaster situations; (ii) a timely COVID-19 Mental Health Action Plan; (iii) inclusion of mental health in the public health agenda; (iv) development of a presidential strategy during the pandemic for comprehensive mental health and well-being; and (v) emerging research assessing the mental health implications of COVID-19.

**Conclusions:** In Chile, the public policy responses to address the mental health consequences of the COVID-19 pandemic has been characterized by the coordinated implementation of mental health plans, ranging from a health sectoral initiative to inter-agency and intersectoral efforts. However, it is imperative that increased funding is allocated to mental health, and efforts should be made to promote the participation of people with lived experiences and communities in the design and implementation of the proposed actions. This aspect could be of key importance to social peace and community recovery after the pandemic.

## Introduction

The coronavirus pandemic, an unprecedented global socio-sanitary crisis, has impacted the mental health of the general population worldwide, and particularly that of individuals with prior mental illness, those infected with SARS-CoV-2, and health workers (1). The exact extent of this impact has varied by country, as it is strongly shaped by local mental health policies that were implemented as part of the initial response to the pandemic (2, 3). The pre-pandemic development of each country's mental health system, and its unique social and economic context, largely define its ability to implement the proposed policies, especially in light of the economic recession that could deepen inequalities in access to and quality of mental health care (4). Under these circumstances, mental disorders co-occur with other chronic diseases that are rooted in social and economic inequities (5, 6). International technical organizations recommend emergency policies that can create safe and dignified conditions for the entire population, by strengthening communities and families, expanding the reach of primary mental health care services, and preserving access to specialized services (2, 7–9). Policies can play a relevant role in mitigating the impact of crises on mental health, either through new initiatives or by deepening health systems reforms, aimed at uplifting communities and increasing user participation (7, 10). Health systems across the Americas often lack resources to develop mental health services in response to crises, which results in an imbalance between the burden of mental disorders and the allocated mental health budget. There is also a wide variation between countries: before the pandemic, it was estimated that this imbalance ranged from 1.8 to 72.1 times the burden of mental illness in relation to spending, with a median of 6.1 in the region (11).

In May 2020, the Americas concentrated the largest number of COVID-19 cases in the world. The first confirmed case in Latin America was registered in Brazil (February 2020), and the first case in Chile was confirmed on March 3rd (12). Chile has a decades-long trajectory of promoting community-based mental health, with mental health services integrated into primary health care centers and general hospitals, in a consistent and sustained way, as outlined in three national mental health plans (from 1993, 2000, and 2017). Before the pandemic, these plans had achieved greater access to community care for people with mental illness, trained mental health workers in the community model, and developed evidence-based, technical guidelines that have improved the quality of care and contributed to a progressive construction of an information system on mental health services. Nevertheless, strategies to encourage user involvement in mental health services have been insufficient (13, 14). Although the public budget is growing, there is a significant gap between what is laid out in the country's mental health plans and the reality of services.

The proportion of the total burden of disease attributable to mental disorders is 9.6 times the proportion of the health budget allocated to mental health (11). The current national mental health plan (2017–2025) includes cross-cutting principles and approaches, such as the respect and promotion of human rights, people-centered health services and equity, evidence-based practices, life-course and multisectoral interventions, and empowerment of persons with mental disorders and psychosocial disabilities. The plan prioritizes seven pillars of action: (i) policy, law, and human rights; (ii) mental health services; (iii) financing the mental health system; (iv) quality, information, and research systems; (v) human resource development; (vi) social participation, and (vii) intersectoral coordination (15).

Chile faced the start of the pandemic in the midst of a profound social and political crisis. In October 2019, a popular uprising emerged, demanding social justice and equity in numerous areas, including health (16). This “Social Outburst,” triggered by secondary students in the face of a rise in the price of the metro ticket, paralyzed the country. Demonstrations in the streets turned violent and questioned the legitimacy of institutions, such as the police, military, and political system. The political crisis gave rise to a referendum for a new constitution, which was postponed because of the pandemic by ultimately passed with overwhelming public support on October 25th, 2020.

Calls for greater equity in access to health services, in particular for mental health, foreshadowed that the impact of the COVID-19 pandemic on mental health; the sanitary crisis has deepened social unrest and increased demands on the health system. For the first time, traditional political opinion polls incorporated questions about mental health (17), and 49.3% of the respondents said that their mood worsened during the pandemic (feelings of rage, sadness, and fear), although 15% perceived that it improved. Toward the beginning of the pandemic, a presidential commission known as the “National Social Committee,” made up of national and local government representatives, health specialists, and academics was formed to “strengthen the country's strategy and organize a single voice in the fight against the coronavirus,” and the Committee incorporated mental health into the national plan to confront the pandemic (18). Given the aforementioned context in Chile, recently implemented mental health policies may play a relevant role in mitigating the pandemic's impact on the population's mental health; however, to do so, these new policies should be coherent with international recommendations and Chile's previously defined plans and policies, while also considering the ongoing scenario of protests and broad social demands.

The international literature has addressed the consequences of the pandemic on mental health, and recommendations or policy papers can be found (19, 20), as well as documents that report the impacts of the pandemic on mental health services (21). One article describes experiences of mental health service response, based on an evaluation of lessons learned by a panel of experts in 16 locations in Australia, Denmark, Italy, Spain, Taiwan, Great Britain, and the United States (22). There is also literature presenting developments in specific countries, such as Rwanda (23), China (24), and Great Britain (25). Similarly, a recent scoping review of how governments, agencies, and organizations responded to the mental health challenges of the pandemic found information on country-specific responses in New Zealand, Australia, China, Finland, India, Iran, Italy, Lebanon, Spain, United States (26). However, there is no literature on government policy and health service responses in Latin American countries that would improve the global learning of other countries and how to drive better local decision-making in mental health care. A “global” approach—can enhance the understanding of how countries are coping with the mental health consequences of the pandemic, taking into account the specific characteristics of the Region and the detrimental effects that the COVID pandemic has caused over other regions of the world. Finally, the experience of Chile, examining how system-level interventions, prevention strategies, mitigation policies, and social responses during the COVID-19 epidemic can improve the knowledge and provision of mental health services in other countries.

This paper aims to critically examine and gather a set of knowledge and lessons learned on public policy in Chile, which can provide evidence and support to vulnerable populations at risk of developing mental health problems in other parts of the world, and heighten the health services demand at this critical period of a global pandemic.

## Materials and Methods

The primary aim of this paper is to describe the public policy responses that have been implemented in Chile to address the mental health consequences of the pandemic during its early stages and compare it with the available experiences of other countries. Two further objectives were (i) to analyze the continuity and discontinuity of this response within the trajectory of mental health policies established before the pandemic, and (ii) to analyze the articulation of this response with international experiences and emerging scientific evidence on aspects of the pandemic that impacts mental health.

The study is based on a general literature review of policies addressing the mental health implications of the COVID-19 pandemic in Chile, including health system interventions to adapt procedures to the context of the pandemic and policies to mitigate the consequences of the pandemic on mental health. We reviewed government-originated responses, as well as those led by civil society organizations that targeted the general population.

### Search Strategy and Selection Criteria

The search strategy was aimed at documents that are publicly available, including scientific literature, official documents and archives, working documents, scientific presentations, press archives, official web pages, and audiovisual archives. The search period ranged from the date of the first COVID-19 case in Chile (March 3, 2020) to the announcement of the “step-by-step” gradual deconfinement plan (July 20, 2020) (27).

Documents were eligible for inclusion if: (i) the population was any population living in Chile; (ii) the intervention was any policy intervention from March 3 to July 20, 2020, that was enacted or implemented by any national government agency, ministry, or government department; and (iii) the outcome measured the consequences of COVID-19 on mental health.

### Data Analysis

Content analysis was used to determine the presence of certain categories within the given qualitative data. Information was organized into four predefined categories and one emerging category. The predefined categories were key actions of the public response (in chronological order): A first response strategy based on a Mental Health and Disaster Risk Management Model; the COVID-19 Mental Health Action Plan; the “National Social Committee;” and, finally, the presidential initiative is known as “SaludableMente.” An emergent category covered research initiatives that directly addressed COVID-19 and mental health. To interpret the results, pre-pandemic mental health policy developments in Chile (objective 2) and international experiences that address the mental health consequences of the pandemic and the scientific evidence published to date (objective 3) was used as a general framework.

All the authors participated in the data extraction and analysis, and the coding process was always verified by at least two authors. Disagreements were resolved by a third author and discussed among the other co-authors.

## Results

### Mental Health and Disaster Risk Management Model

Chile began developing a Mental Health Care and Disaster Risk Management (MHCDRM) Model in 2018, in collaboration with Japan. The evidence-based Model highlights national experience gleaned through major natural disasters that have affected Chile, and it complies with international humanitarian standards. Essential elements of the Model include reducing vulnerability through strengthening community resilience and capacities and focusing on preventive, rather than reactive, interventions. It proposes the implementation of mental health and psychosocial support (MHPSS) actions throughout the risk management cycle and effective disaster risk reduction, adopting the integration of interventions at different levels, according to the Inter-Agency Standing Committee's (IASC) recommendations (2). The MHCDRM Model is organized in eight strategic pillars—(i) intersectoral coordination; (ii) information management; (iii) social communication; (iv) community empowerment; (v) education; (vi) focus on vulnerable groups; (vii) technical guidelines; and (viii) care for frontline workers—and has led to the implementation of intersectoral mental health and psychosocial support committees and the development of a psychological first aid (PFA) training plan, which has a network of over 900 trainers and has produced more than 10,000 people qualified to provide PFA throughout the country.

Though the Quintero-Puchuncaví socio-environmental conflict and the social uprisings that recently affected the country were very different types of crises, they both effectively used the MHCDRM Model. In both emergencies, mental health was included in the first line of response, for the very first time, and the Model was relevant to define and organize pertinent actions. This Model has also been used as a referential framework to implement strategies to protect mental health during the COVID-19 pandemic, including strategies, focused on providing mental health support and responding to the psychosocial needs of specific groups that are in greater biopsychosocial vulnerability (28).

### COVID-19 Mental Health Action Plan, Headed by the Ministry of Health

To articulate and organize multiple interventions to protect mental health during the COVID-19 pandemic, an Action Plan on Mental Health was developed by the Ministry of Health. The Plan includes seven areas of action (Table 1): (i) Continuity of care and strengthening of mental health services; (ii) Intersectoral coordination; (iii) Specific populations; (iv) Care of the healthcare workforce; (v) Community strengthening and social communication; (vi) Information management; and (vii) Training and technical guidelines for the intervention.

**Table 1:** COVID-19 Mental Health Action Plan: areas of action, objectives, and strategies.

**General objective**
Strengthen capacities to mitigate the impact on the population's mental health in the context of the COVID-19 pandemic, through the adaptation of mental health services, intersectoral coordination, and targeting of groups with the greatest biopsychosocial vulnerabilities.
**Area 1: Continuity of care and strengthening of mental health services**
Objective: Strengthen contingency strategies in the public health network to address mental health and safeguard access, opportunity, and continuity of mental health care in primary care centers, an outpatient services, hospitals, and residential facilities.
Strategies: · Adaptation of the health network for the care of people with mental health needs. · Support for the continuity of mental health actions at the regional level (SEREMI). · Delivery of mental health recommendations in the context of the COVID-19 pandemic. · Optimization of resources to maintain essential functions and the management of complementary resources in the mental health network. · Protection of the management of the mental health network for addressing mental health problems and ensuring continuity of care for people at high risk of suicidal behavior, decompensated psychosis, and behavioral disorders.
**Area 2: Intersectoral coordination**
Objective: To facilitate the articulated and synergistic action of multiple public actors and civil society in the approach to mental health and psychosocial support in the context of COVID-19.
Strategies: · Constitution of a network of remote helplines. · Cooperation between intersectoral agencies and organized civil society. · Installation and operation of a National Intersectoral Technical Coordination Table.
**Area 3: Specific populations**
Objective: Reduce the mental health risk of specific groups that are in conditions of greater biopsychosocial vulnerability during the pandemic, through timely and pertinent actions relevant to their particular characteristics and needs.
Strategies: · Delivery of recommendations for the implementation of preventive actions, early detection, and treatment of mental health problems and/or diseases in populations directly affected by COVID-19. · Implementation of actions that promote access to mental health and psychosocial support services in the context of COVID-19, which are pertinent and timely for people at greater risk of discrimination, violence, and social exclusion. · Development of technical guidelines aimed at caregivers of people in situations of disability and/or dependency, aimed at reducing risk factors and promoting protective factors for mental health, for themselves and the people under their care. · Technical support for the implementation of actions in the context of COVID-19 for the promotion of the psychosocial well-being of people living in residential facilities and in contexts of institutionalization.
**Area 4: Care of the healthcare workforce**
Objective: To mitigate the impact on mental health of the health workforce in the context of COVID-19, through the promotion of self-care, mutual care, and institutional care.
Strategies: · Remote psychological support systems for health personnel from the Digital Health Department. · Institutional Care of Health Personnel through the development of local action plans and support resources.
**Area 5: Community strengthening and social communication**
Objective: To promote social support and mobilization of the communities' own resources for the mitigation and reduction of psychosocial risk factors resulting from the pandemic through mechanisms of community participation, interaction, and social organization, and through strategies for social communication and education for the protection of mental health.
Strategies: · Preparation and dissemination of communication materials for the population. · Guidelines for the design and implementation of education and social communication strategies aimed at the population. · Support for collaborative work with community-based organizations, community leaders, and stakeholders, to facilitate actions of social support and mutual care. · Promote community participation in detecting mental health needs and proposing territorial actions.
**Area 6: Information management**
Objective: To establish mechanisms to have valid and timely information for informed decision-making, including diagnosis, systematization, analysis of information, and monitoring, that allow efficient actions to be taken to protect mental health during COVID-19, in both sectoral and intersectoral networks.
Strategies: · Development of systems that allow monitoring of the actions carried out by the health network. · Implementation of a system for registering user follow-up actions. · Systematic monitoring of the situation of the Mental Health Network. · Support the dissemination of experiences and practices of the network and other sectors. · Generation of information exchange mechanisms at the sectoral and intersectoral levels.
**Area 7: Training and technical guidelines for the intervention**
Objective: To develop technical guidelines for mental health protection and psychosocial support actions during COVID-19, based on available evidence and national and international standards, as well as instances for their technical transfer to healthcare workers and teams.
Strategies: · Preparation of a technical reference framework for the protection of mental health and psychosocial support in the context of COVID-19. · Preparation of thematic guidelines for the work of health teams and psychosocial teams in the context of COVID-19. · Preparation of guidelines for remote mental health care strategies for the health network. · Adaptation of the First Psychological Aid model to the context of COVID-19. · Development of dissemination strategies, technical transfer, and training of technical guidelines during COVID-19. · Collaboration in technical transfer and training processes developed by other sectors and civil society organizations.

*Based on Ministry of Health data*.

As part of the implementation process of the Plan, mental health care in primary health centers and outpatient specialty services were improved (29), and mental health services were incorporated into the rural and remote health care facilities. Additionally, inpatient psychiatric services were adapted to meet COVID-19 protocols, registration systems were updated, and an online monitoring system for the mental health network was developed.

Another achievement was the organization of a Mental Health Personnel Commission in the Ministry of Health, which recommended the implementation of a nationwide institutional care program with psychological support strategies for healthcare workers (30). To support this process, technical recommendations were distributed (31).

Furthermore, online and telephone support services were made available for health workers and the general population. With over 100 helplines from academic and civil society initiatives, a national registry was built, to strengthen technical capacities and coordinate actions, establishing referral flowcharts, and management protocols. These developments are detailed in two bulletins that provide information on remote mental health helplines and psychosocial support in the context of COVID-19 (32, 33).

Another relevant initiative within the framework of the Plan was the organization of webinars and teleconferences, as an education and training strategy, targeting the workforce of health and social programs, to improve their preparation to provide psychosocial support for COVID-19 patients and their families. To date, 13 webinars have been developed, in partnership with the School of Public Health of Universidad de Chile and the Department of Mental Health of the Ministry of Health, covering topics such as outpatient and inpatient mental health care, mental health for the elderly, mental health for children and adolescents, alcohol and other drugs prevention, and the mental health of health care workers (34). Presenters and trainers included advisors from the Ministry of Health and researchers and mental health teams working in Chile, Peru (Huánco), and Spain (Madrid and Valencia). In addition, a series of online training resources, including remote help and psychological first aid tools and open teleconferences on mental health topics, have been implemented (35, 36).

The framework set forth by the Mental Health Action Plan during COVID-19 continues to support the organization, implementation, and monitoring of public policy responses to the pandemic.

### National Social Committee: Mental Health Strategy on the Political Agenda

The National Social Committee worked on a national strategy for mental health, formulated by researchers and academics from the Universidad de Chile. Their proposal was subsequently enriched with the contributions of other committee members and academics from other universities. The final Strategy included mental health guidelines from the Ministry of Health and recommended adopting an intervention pyramid to provide mental health and psychosocial support during emergencies (2). As such, mental health became a part of the national pandemic response. The Strategy called for the protection of individuals who were most vulnerable to experiencing mental health crises during and after the pandemic, and it declared that efforts should not be limited to simply providing intensive care in hospitals. The main message was that “mental health is one of the keys to surviving this pandemic and all that it entails in the short, medium, and long term, from preventing a potential crisis in the provision of health services, to preserving and rebuilding a post-pandemic society” (37).

The Strategy includes three goals: (i) to reduce population risk by strengthening psychosocial protective factors for mental health; (ii) to facilitate access to comprehensive, equitable, and quality mental health services; and (iii) to develop knowledge, practices. and mental health competencies among mental health workers. The document states that mental health policy must meet four criteria: (i) territorial articulation; (ii) intersectoral action; (iii) user involvement and participation; and (iv) economic, social, and human development. This statement emphasizes the need to conduct community-based interventions, and work with social institutions, to avoid reducing mental health problems to an individual level. The mental health policy thus conceived a “comprehensive perspective, without prioritizing economic factors over social and human ones,” to respond to the pandemic.

### SaludableMente^1^ Initiative: the Presidential Strategy on Mental Health

On May 17, 2020, during a nationwide television broadcast, President Sebastián Piñera announced the creation of the Healthy Mind Initiative (Iniciativa SaludableMente) whose goal was “to improve the public and private mental health services in [Chile].” SaludableMente is defined as a “comprehensive pandemic response plan for mental health and well-being,” which includes two pillars: (i) a digital mental health platform and (ii) an experts committee.

The digital platform (38), created to immediately strengthen mental health services, houses all the current programs that promote the mental health and the emotional well-being of different priority groups, including children and adolescents, older adults, parents and caregivers, women who are victims of violence, and individuals with COVID-19, as well as the general population. The platform provides direct access to remote psychological support, which has been integrated into the geographical network of services to improve the continuity of care for patients with mental disorders.

At the same time, the initiative convened a panel of experts to develop proposals and guidelines to respond to the mental health needs of the population during the pandemic (39). The Healthy Mind Committee was officially established and convened its first meeting on June 1, with a period of 90 days to fulfill its mandate. Over thirty representatives were invited to form part of the Committee, including academic experts, representatives of scientific societies and other civil society organizations, members of Congress, and representatives of different ministries. The Committee's first task was to review and expand the Ministry of Health's diagnosis of the mental health situation in the context of COVID-19. From there, working groups were formed on specific topics. Each group developed a roadmap that includes a summary of the current situation, actions, expected results, monitoring activities, and a timeframe, to create an integrated strategy with clear deadlines.

SaludableMente generated a broad, intra-sectoral dialogue that guided government actions around the well-being and mental health of the population, beyond health services. This strategy is still ongoing but has already managed to give greater visibility to mental health, secure new resources, and facilitate the articulation of different perspectives and capacities.

### Emerging Research in Mental Health and COVID-19

The academic community has also reacted quickly to the pandemic, and several research projects related to its mental health effects are underway. Initially, in light of the urgency of the situation, these projects began functioning without public funds, and most of them were based out of academic institutions or programs. Later on, however, the National Research and Development Agency (ANID, for its acronym in Spanish) developed a funding scheme called “Competition for the Rapid Allocation of Resources for Research Projects on Coronavirus (COVID-19),” that financed year-long projects, for a maximum of 90 million Chilean pesos (~USD 118,000), in the broad spectrum of COVID-19 diagnosis, control, prevention, treatment, monitoring, and other aspects related to the pandemic and its consequences, across scientific, technological, health, social, economic, cultural, and humanistic fields (40).

From a total of 1,056 submitted project proposals, 63 projects (6.6%) were awarded funding. Although several projects touched on psychosocial aspects—such as intra-family violence or research on behaviors or attitudes—only three of the awarded projects directly addressed mental health. Although ANID's funding source (the Ministry of Science and Technology) is not considered as part of the health budget (overseen by the Ministry of Health), the small proportion of projects awarded projects to mental health topics is an expression of the same phenomenon of imbalance between health and mental health, that is seen in the small proportion of the Ministry of Health budget dedicated to mental health.

The three financed projects deal with the impact of physical distancing measures on subjective well-being, the impact of the pandemic on the psychosocial well-being of older people, and the evaluation of anxiety and depressive symptoms and risk behaviors during this crisis (40).

Within the 147 projects that were included in the waiting list for the ANID grant, there are 8 projects related to mental health or subjective and psychosocial well-being. There is no information about whether these projects will be carried out or not, but their focuses cover research areas of local interest, including the mental health of the adolescent population, the evaluation of depressive symptoms related to severe COVID-19, the mental health of survivors of intensive care, the psychosocial well-being of migrant children, the mental health of health workers, the use of technologies for psychoeducation, suicide prevention, and post-COVID-19 mental health (40).

In terms of pandemic-focused research that has been developed in Chile, apart from the ANID financing system, one project that is notable for its magnitude and international scope is the COVID-19 HEalth caRe wOrkErS (HEROES) study, aimed at understanding the impact of the pandemic on the mental health of health care workers. This is a prospective cohort study that considers an initial evaluation and three follow-up evaluations (3, 6, and 12 months), led by the School of Public Health of the Universidad de Chile, and which is being implemented in over 30 countries, including The United States, Mexico, Spain, Italy, South Africa, China, and Australia (41).

According to its mid-June progress report, 1,177 health workers had been included in Spain, and 1,213 in Italy. Preliminary results show that 57.3% of workers report sleeping problems, 65.6% have frequently felt emotionally overwhelmed, 51.5% have depressive symptoms, and 35.3% have acute stress symptoms. In most of the sample (71.6%), the participants' scores indicate a “possible case” of anxiety or depressive disorder, while 7.2% have had suicidal thoughts, and 1.7% have had suicidal attempts (within the last few weeks) (42). The study is under development, and these initial trends have yet to be validated.

## Discussion

In Chile, five lines of action in mental health policy were included in the pandemic response. First, a pre-existing Mental Health Care and Disaster Risk Management Model that acknowledged the importance of preparedness to reduce vulnerability and negative outcomes at both the individual and community levels. Second, a COVID-19 Mental Health Response Plan, led by the Ministry of Health, that seeks to meet the population's mental health and wellbeing needs to reduce the negative impacts of the pandemic in the short and long term. Third, the establishment of the National Social Committee, to ensure effective governance, intersectoral coordination, and implementation of mental health policies as part of the response to the pandemic. Fourth, partnerships and collaborations across health services, universities, and other sectors through SaludableMente enabled the optimum use of resources to deliver cohesive and coordinated care and support, although the participation of people with lived experiences and caregivers was very limited. Fifth, research support generates a local body of evidence around the impact of the pandemic on mental health.

These policies are characterized by a coordinated implementation of the mental health plan, from health system initiatives to inter-agency and inter-sectoral work, that included mental health in the national pandemic agenda. Decades of dedicated intersectoral collaborative work, and the trajectory of past mental health policies, has paved the way for mental health to be accepted into the mainstream political dialogue and included in the pandemic response, a recognition that has not been achieved since the first national mental health plan in 1993. However, the pandemic has also revealed areas that need to be urgently addressed and exposed cracks in our already fragile mental health system.

Starting in the early 1990s, after the end of civic-military dictatorship, Chile has set a clear path toward a community-based mental health system, with the following national mental health plans, in 2000 and 2,917, consolidating this transition (15, 43, 44). The gradual deinstitutionalization of former psychiatric inmates, the development of territorialized mental health care services, and the integration of these services in the larger health system are key achievements in the implementation of a community-based model in mental health in the country (13).

This groundwork, and its achievements, have proved useful to the adequate deployment of emergency response strategies. On the one hand, the existence of a coherent network of services at primary, secondary, and tertiary care levels facilitates the provision of mental health services that are more connected to local contexts and needs. At the same time, this prior set of aims—expressed and organized in the latest mental health plan—has provided a strong and practical foundation: The Mental Health Strategy of the National Social Committee, the Mental Health Action Plan, and the SaludableMente Initiative follows the basic principles and structure of the National Mental Health Plan 2017–2021 which, in turn, follows the foundational community-based model. The model provides a shared language across the mental health field.

Nonetheless, two key weaknesses in the transformation of services in the country have also expressed themselves in the mental health response toward the pandemic. The first weakness is the low budget allocated to mental health (45) in the country, a decades-long “debt.” This impacts the ability to increase direct mental health services in the face of the growing demands of the population due to the pandemic. It also narrows the margin of actions that can be carried out beyond clinical care, such as community strengthening and directly supporting grassroots, bottom-up forms of services that have nonetheless emerged during the crisis.

Directly related to this, Chile's second historical debt is the lack of participation of local communities and especially of mental health service users (14, 46). COVID-19 related mental health policy has been developed without the participation of user groups. The SaludableMente initiative, while giving a place for experts and other civil society groups, did not incorporate user organizations in its original design, despite the existence of such groups in the country. On the other hand, the current mechanisms of participation set in place in the healthcare sector have no binding power and are therefore limited in their ability to effectively influence policy (47).

Despite having a community-based model, over the last 30 years, mental health policymaking in Chile has largely considered communities to be passive settings where clinical services are provided. The socio-political agitations of 2020, with the “social outburst” and the current sanitary crisis have, nonetheless, turned mental health into a public concern, and the political system is only just reacting to this. Chile faces the coming challenge of deepening its original, democratic vocation and establishing solid forms of participation for users and communities. This will be especially critical to regain the health system's full capacity to address the population's mental health needs.

The mental health policies that were implemented in Chile during the pandemic are concordant with international recommendations and the available scientific evidence in various aspects, from the adoption of a humanitarian response model for socio-health emergencies to the identification of numerous target populations, including: (i) the general population, due to the risk associated with quarantine measures and confinement; (ii) those with pre-existing mental illnesses, due to the risk of exacerbation of symptoms, obstacles to treatment continuity, and discrimination in care due to COVID-19; (iii) the survivors and the families of victims, due to the trauma of contagion and the experience of mourning; and (iv) health workers, due to the risk of burnout and stress. The Chilean policies are also in line with leading recommendations that mental health services should be organized according to a community model, with priority given to strengthening primary care; that efforts should be made to ensure the continuity of care for mental health services users, by incorporating remote care and providing personal protection elements to community-based teams; and that intersectoral policies should be formulated. The previous experience in the country and the development of such an approach explain the rapid organization in Chile of mental health planning to address the pandemic, incorporating various health policy governance strategies, including the priority assigned by the presidency and the intersectoral participation. This appears to be a differentiating element, compared to the initial response described for other countries, which is more focused on service management.

Nevertheless, certain aspects of international recommendations and scientific evidence have not been sufficiently developed in Chile: (i) the focus on health rights (although this principle is included in the 2017–2025 National Mental Health Plan); (ii) the participation of users and their families; (iii) the neuropsychiatric effects of COVID-19 survivors; (iv) the impact of the pandemic-related economic recession on mental health; and (v) the amount of funding required or allocated to mental health. The lack of resources and preparedness are common in many countries dealing with the consequences of an unprecedented scenario (22). The mental health approach to emergencies and disasters, well developed in Chile due to long exposure to natural disasters—including trained technical and human resources—could have compensated for the lack of mental health funding and specific technical preparedness for the COVID-19 pandemic.

It is also possible to observe in other countries and regions the primordial need to develop rapid response strategies to mental health crises as a consequence of the pandemic, adapting services to ensure continuity of care and access to care for the new demand that has arisen (22). Of particular relevance during the pandemic seems to be the development of online and telephone support strategies, such as those compiled by McCartan et al. (26) for Afghanistan, Egypt, Iran, Iraq and Palestine, as well as, the establishment of an online portal to address mental health issues, such as the one described by the same authors in The Netherlands. The Chilean experience can well be placed within these frameworks.

The emerging idea—from a review of pandemic coping in several countries—that the crisis offers some opportunities, such as intersectoral collaboration and increased investment (26), is consistent with what has been experienced in Chile. However, we also agree that even greater changes are required to address the social inequalities that are at the heart of the mental health consequences of the pandemic.

### Limitations

The authors are policy stakeholders, either from the Ministry of Health or from the School of Public Health of Universidad de Chile, and they acknowledge that the risk of bias could be a limitation in the execution of the study. To address this potential bias, only publicly available documents were selected, so that they could be analyzed, evaluated, and interpreted by readers of this manuscript and other interested parties.

## Conclusion

In this paper, we have described the main aspects of the policy responses to the mental health challenges derived from the COVID-19 pandemic in Chile, analyzing their integration within the pre-pandemic mental health policy framework and service trajectory, and comparing the policies with available evidence and experiences from other countries and international agencies, such as the World Health Organization. Using available local materials developed during the pandemic, we have identified five key elements, consisting of four broad policy initiatives and research efforts based out of local universities and institutions.

In light of the multiple strengths of the Chilean response to mental health challenges during the pandemic, it is surprising that a community participation approach was not observed. This discrepancy reflects a tension, between a response focused on intensive hospital beds vs. initiatives that strengthen communities and non-specialized mental health services. This tension was noted in the mental health strategy document that was provided to the National Social Committee, and it is a priority policy area that Chile has worked on developing in recent national mental health plans. To face the predicted post-pandemic increase in demand for mental health services, priority resource allocation must consider community settings.

The pandemic, however, is far from over, and its impact upon the population's mental health will continue to be revealed over time. As such, the effects of these mental health policy responses in the real world have yet to be estimated. What is clear is that these initiatives need to be documented, evaluated, and shared to create a pool of experiences that can guide other countries in this process. In Chile, the pandemic has created opportunities to increase policy action in mental health, and this can also be the case for our regional neighbors. Positioning the community mental health model as a framework for pandemic policymaking, the government-led mental health response could contribute to social peace and play a key role in building a post-pandemic Chilean society. For this reason, citizen participation will be of key importance. The pandemic could be considered the greatest current challenge of mental health policy in Chilean modern history, and the principles and visions outlined in the National Mental Health Plan 2017–2025 should be urgently implemented in the actual practice of health services.

## Author Contributions

MI, PN, CM, OT-D, BV, and AC-U contributed to the design and implementation of the research, to the analysis of the results, and to the writing of the manuscript. All authors contributed to the article and approved the submitted version.

## Conflict of Interest

The authors declare that the research was conducted in the absence of any commercial or financial relationships that could be construed as a potential conflict of interest.

## Publisher's Note

All claims expressed in this article are solely those of the authors and do not necessarily represent those of their affiliated organizations, or those of the publisher, the editors and the reviewers. Any product that may be evaluated in this article, or claim that may be made by its manufacturer, is not guaranteed or endorsed by the publisher.

## Footnotes

^1^“SaludableMente” in Spanish means Both “Healthy Mind” and “Healthily”.
